# Supplementary material for: Genetic Biosensor for Optimizing Double-Stranded RNA Production by Bacteria
Source: ACS Synth Biol. 2026 Jul 6;15(7):2812–6. doi: 10.1021/acssynbio.6c00080 (PMC13386644; doi:10.1021/acssynbio.6c00080)
Supplement: Supplementary file 2 [file sb6c00080_si_002.pdf]

Genetic Biosensor for Optimizing Double-Stranded RNA Production by Bacteria

Lucio Navarro-Escalante[1*], Anthony J. VanDieren[2], Jeffrey E. Barrick[1,3*]

[1]Department of Microbiology, Genetics, & Immunology, Michigan State University, East Lansing, Michigan 48824, United States. [2]Department of Molecular Biosciences, The University of Texas at Austin, Austin, Texas 78712, United States. [3]Department of Entomology, Michigan State University, East Lansing, Michigan 48824, United States.

*Correspondence: L. N.-E. (lucionavarro@cafedecolombia.com) and J.E.B. (jbarrick@msu.edu)

[†]Current Address: Department of Entomology, National Coffee Research Center (Cenicafé), Manizales, Caldas 170009, Colombia.

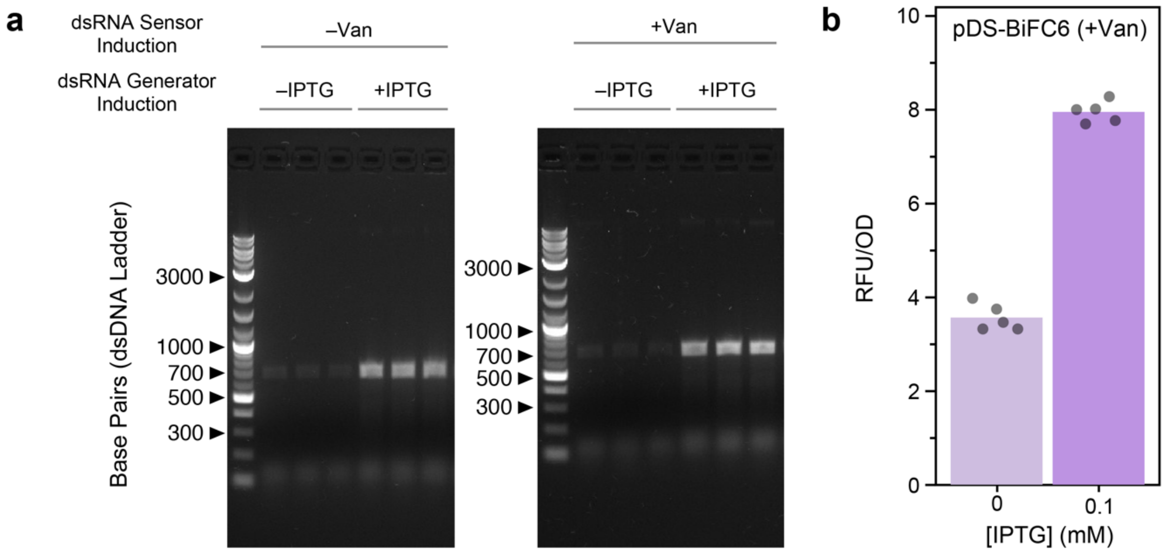

Figure S1. **Validation of dsRNA production**. *Escherichia coli* HT115 (DE3) cells transformed with the IPTG-inducible dsRNA generator plasmid pDS-GFP<T7 and the Van-inducible dsRNA sensor pDS-BiFC6 were diluted from overnight cultures to an OD600 of 0.2 into fresh media and incubated for 4 h of growth. Five replicate cultures were tested with every combination of the presence and absence of the two inducer molecules. (**a**) dsRNA purified from equal volumes of three of the five cultures in each group visualized on agarose gels. The heterologous GFP dsRNA expressed from pDS-GFP<T7 migrates at ~700 bp relative to the dsDNA ladder. Its abundance increased when IPTG was added. (**b**) YFP RFU/OD600 measured for the same cultures in which the dsRNA sensor was induced with Van. Signal from the dsRNA sensor in these cells approximately doubled when dsRNA production was induced with IPTG.
